# BMP Signaling and the Maintenance of Primordial Germ Cell Identity in Drosophila Embryos

**DOI:** 10.1371/journal.pone.0088847

**Published:** 2014-02-14

**Authors:** Girish Deshpande, Elinor Willis, Sandip Chatterjee, Robert Fernandez, Kristen Dias, Paul Schedl

**Affiliations:** 1 Department of Molecular Biology, Princeton University, Princeton, New Jersey, United States of America; 2 Institute of Gene Biology RAS, Moscow, Russian Federation; National Cancer Institute, United States of America

## Abstract

The specification of primordial germ cells (PGCs) and subsequent maintenance of germ-line identity in *Drosophila* embryos has long been thought to occur solely under the control of cell-autonomous factors deposited in the posterior pole plasm during oogenesis. However, here we document a novel role for somatic BMP signaling in the maintenance of PGC fate during the period leading up to embryonic gonad coalescence. We find that PGCs fail to maintain their germline identity when BMP signaling is compromised. They initiate but are unable to properly assemble the germline stem cell-specific organelle, the spectrosome, and they lose expression of the germline-specific gene Vasa. BMP signaling must, however, be finely tuned as there are deleterious consequences to PGCs when the pathway is excessively active. We show that one mechanism used to calibrate the effects of BMP signals is dependent on the Ubc9 homolog Lesswright (Lwr).

## Introduction

The embryonic gonad of *Drosophila* consists of two cell types, the somatic gonadal precursor cells (SGPs) and the primordial germ cells (PGCs). The SGPs are specified by zygotic genes that pattern the mesoderm during mid-embryogenesis [1]. In contrast, PGCs are specified at the syncytial blastoderm stage by a mechanism that is thought to depend exclusively on maternal determinants that are localized in the posterior pole plasm during oogenesis [2]–[4]. Nuclei migrating into the pole plasm undergo precocious cellularization, incorporating the maternal determinants [5]. These determinants are thought to be both necessary *and* sufficient to program the newly formed pole cells to assume and then maintain a distinct PGC identity [3], [4]. Instead of continuing to divide like the neighboring somatic nuclei, the PGCs divide once or twice and arrest in G2. Also unlike somatic nuclei which upregulate Polymerase II activity during the mid-blastula transition, the PGCs enter a state of transcriptional quiescence. The downregulation of transcription is critical for the proper specification of PGC identity [3] [4] [6]. In addition to programming the germline transcriptome, transcriptional quiescence is also thought to protect the PGCs from the effects of signals emanating from the surrounding soma [6]. During gastrulation, the PGCs are carried inside the embryo, and zygotic transcription begins in the PGCs at stage 9 [7]. Between stages 10–13 they migrate through the midgut epithelium and then move dorsally along its basal side toward two groups of SGPs in the mesoderm [8]. 10–15 PGCs contact each group of SGPs at stage 13. Finally, the SGPs and PGCs coalesce to form the primitive embryonic gonad.

Although the idea that *Drosophila* PGCs are refractory to the effects of somatic signaling molecules during early embryogenesis has now been widely accepted, there is, in fact, evidence that these cells have the machinery needed to perceive somatic signaling molecules. One of these signaling molecules is the Bone Morphogenetic Protein (BMP) Decapentaplegic (Dpp). Studies by Dorfman and Shilo [9] showed that blastoderm stage PGCs have high levels of the BMP pathway transcription factor Mothers Against Dpp (Mad) and that this protein is phosphorylated and translocated into the nucleus in response to BMP signals from surrounding soma [9]. This is most clearly demonstrated by the complete absence of nuclear pMAD in PGCs of mutant embryos that do not express *dpp.* Further supporting the conclusion that newly formed PGCs express and utilize the canonical BMP signal transduction machinery, Dorfman and Shilo [9] found that the gene encoding the BMP receptor *thickveins* had to be mutant in both the mother’s germline and in the zygote in order to block pMad accumulation in PGC nuclei.

Of course, the fact that PGCs are able to perceive BMP signals from the nearby soma does not mean that they are capable of responding to this signal or, if they do, that the response is important for PGC development. In fact, since the PGCs are transcriptionally quiescent, a plausible assumption has been that this nuclear pMad is simply inert, unable to exert any regulatory effects on its target genes. On the other hand, in the adult gonad, BMP signaling from the soma plays a critical role in the self-renewing properties of the descendents of the PGCs, the germline stem cells (GSCs). The GSCs reside at the tip of the adult male and female gonad in a special somatic microenvironment called the niche [10]–[12]. BMP signals from this niche ensure that when GSCs divide, they do so asymmetrically so that the daughter cell closest to the niche and the source of the BMP retains GSC identity [13], [14]. When the BMP signaling pathway is compromised, GSCs lose the ability to self-renew and enter the gamete differentiation pathway. Conversely, excess BMP signaling induces GSC over proliferation [13].

The fact that BMP signaling plays such a central role in maintaining GSC identity prompted us to ask whether this signaling pathway also impacts the development of the PGCs in the embryo. Here we have focused on the period after the PGCs exit the mid-gut, migrate towards the SGPs and then coalesce into the embryonic gonad. During this period, the PGCs begin to reactivate transcription and start assuming some of the morphological features characteristic of the GSCs in the adult gonad. Our studies show that BMP signaling during this period in mid-embryogenesis is critical for the maintenance of PGC identity. However, the response to external BMP signals must be delicately adjusted, as excess signaling has deleterious effects on the PGCs. We show that a component of the sumoylation pathway, *lesswright* [15], [16] functions in PGCs to dampen the response to BMP signals from the soma.

## Results

### PGC-specific Nuclear Accumulation of pMad Follows a Dynamic and Stage- Specific Pattern

**Figure 1:**
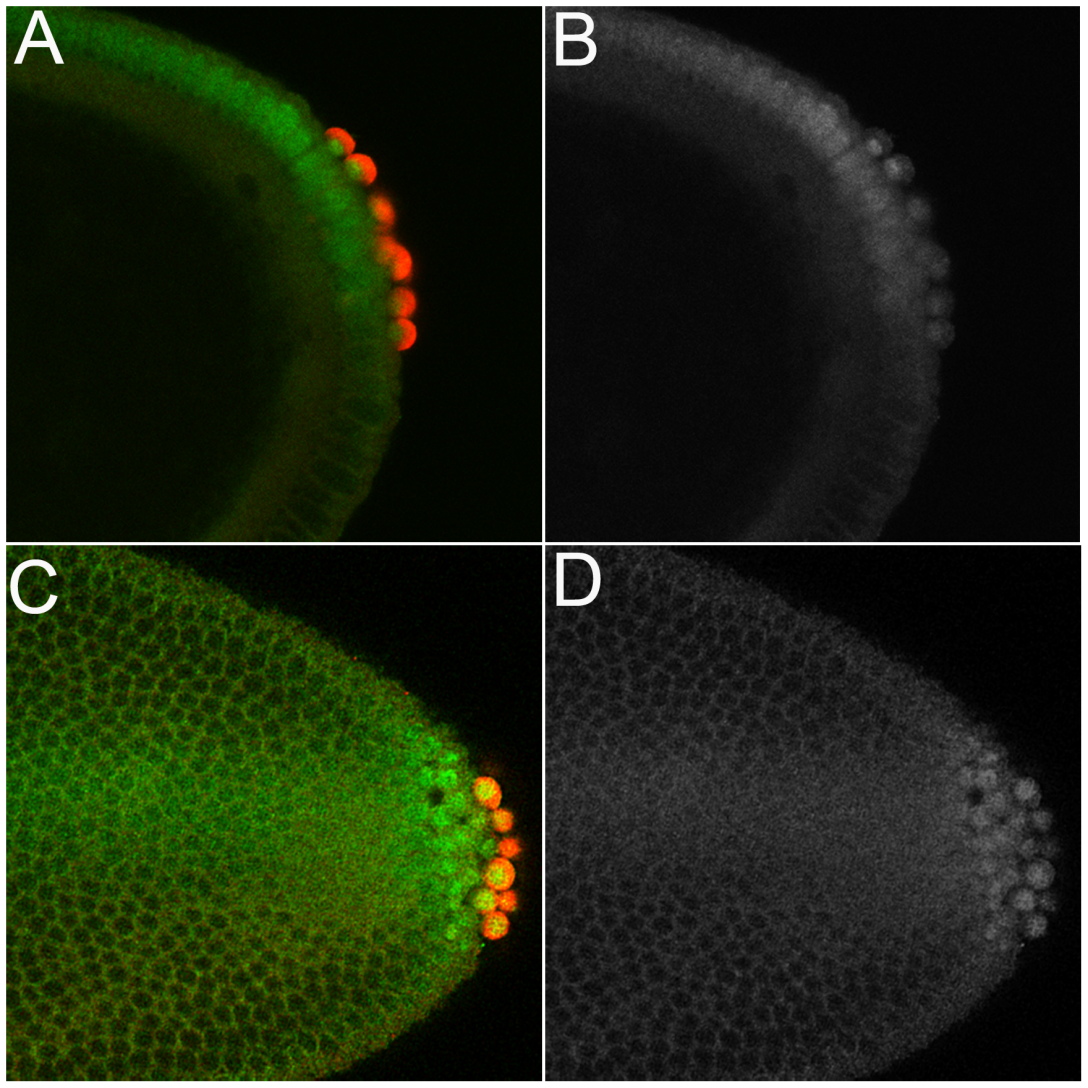
pMad is enriched in early pole cell nuclei. **A, B.** Stage 5 wild type blastoderm stage embryo shows pole cells (with nuclear accumulation of pMad. Dorsal somatic nuclei also show high levels of nuclear pMad. **C, D.** Stage 6 wild type embryo also shows high nuclear pMad levels in the pole cells and dorsal somatic cells. Panels A and C: Vasa imaged in red; pMad imaged in green. Panels B and D: gray scale image of pMad.

**Figure 2:**
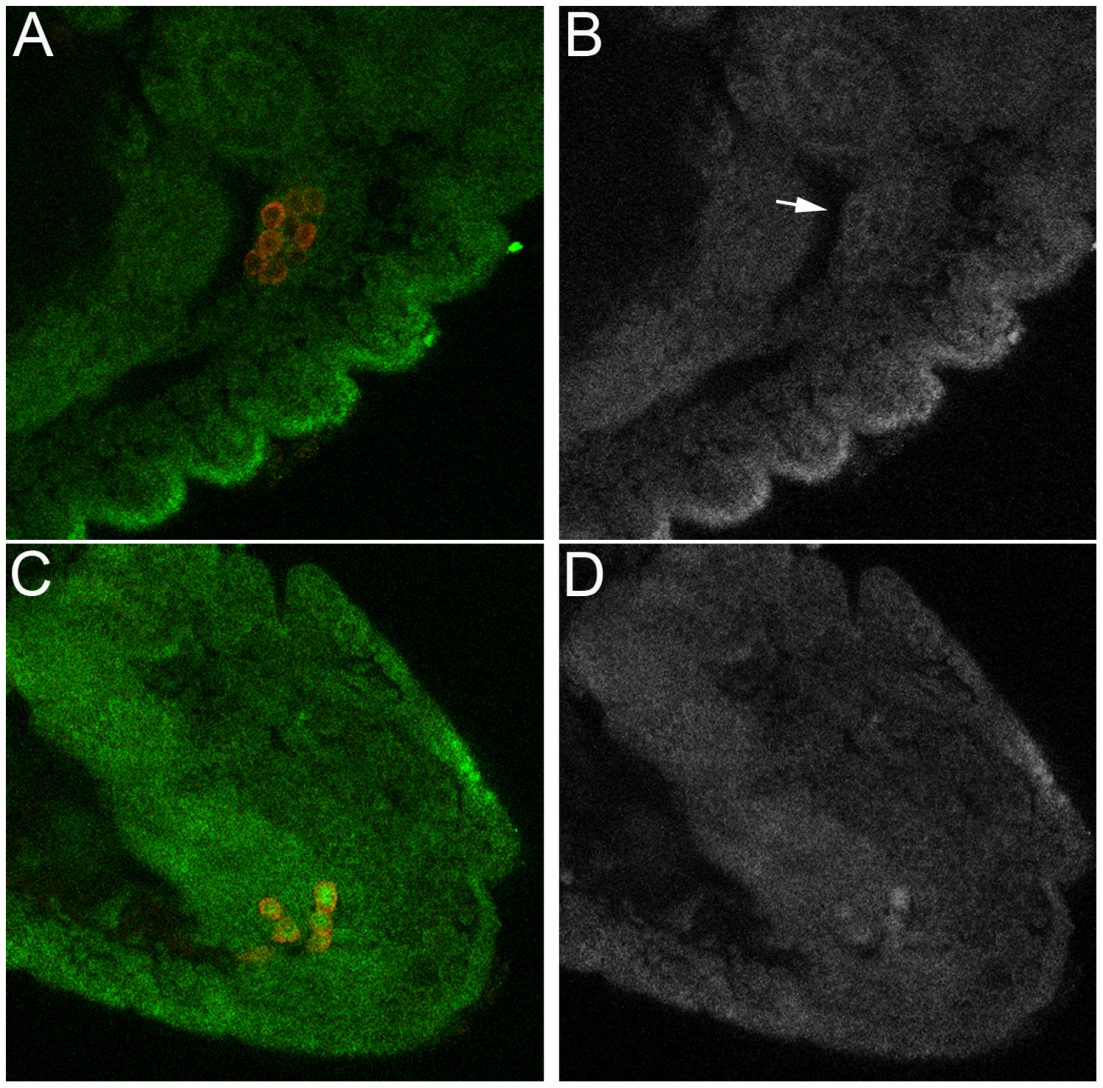
Mid-stage PGCs can respond to Dpp signaling. **A, B.** Stage 12 wild type embryo. **C, D.** Stage 12 *twi-Gal4/+;UAS-dpp/+* embryo. Panels A and C: Vasa imaged in red; pMad imaged in green. Panels B and D: gray scale image of pMad. In wild type (A, B) nuclear pMad levels are near background; however there does seem to be some cytoplasmic protein in PGCs (seen as a ring around each PGC nucleus: see arrow in panel B). In *twi-Gal4/+;UAS-dpp/+* embryos nuclear pMad is readily detected (see panel D). Dpp expressing embryos were generated by mating virgin females carrying two copies of the *twi-Gal4* driver with males carrying two copies of *UAS-dpp.*

**Figure 3:**
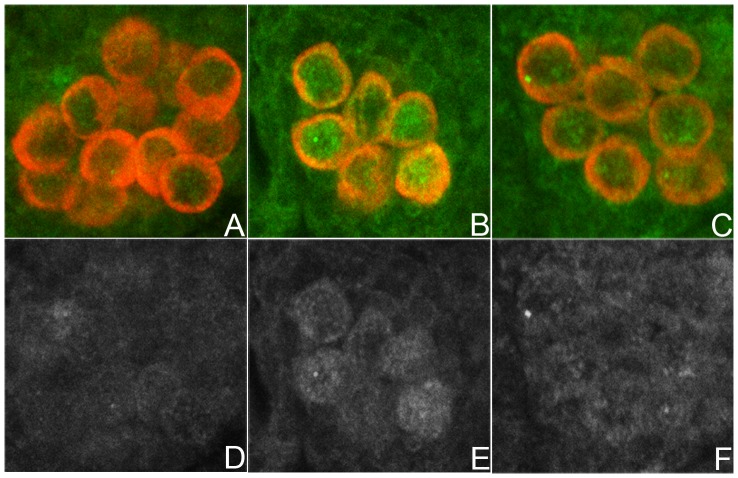
pMad accumulation in stage 15 PGCs. **A–C.** Stage 15 embryos stained for Vasa (red) and pMad (green). **D–F.** Grey scale images of the same embryos showing only pMad. A,C: Wild type gonad. B,E: *nos- Gal4/UAS-dpp* PGCs. Levels of nuclear and cytoplasmic pMad increase in PGS when Dpp is overexpressed. C,F: *nos-Gal4/UAS-lwrDN* PGCs. Levels of nuclear and cytoplasmic pMad increase when LW activity is inhibited. All embryos carry a single copy of the driver and a single copy of the *UAS* transgene.

Dorfman and Shilo [9] showed that blastoderm stage PGCs have high levels of nuclear pMad. We confirmed this observation (Fig. 1) and then followed pMad accumulation in PGCs from this stage through the formation of the embryonic gonad. We found that nuclear pMad persists until the PGCs are carried into the interior of the embryo during gastrulation and become refractory to detailed analysis. Strikingly, when the PGCs emerge from the midgut at stage 10 and begin to move along its outside surface, only little nuclear pMad is observed. pMad remains at low levels through stages 12–13 (Fig. 2A and B) while the PGCs are migrating towards the SGPs. pMad levels then increase in PGC nuclei when the gonad coalesces during stages 14–15 (Fig. 3A). However, the pattern of pMad accumulation in the PGCs of the newly coalesced gonad differs in two important respects from that seen in blastoderm stage PGCs. First, the overall level of nuclear pMad in stage 14–15 PGCs is lower than that in blastoderm stage PGCs (compare Fig. 1 with Fig. 3A). Second, while pMad in the blastoderm stage PGCs is largely nuclear as expected, much of the pMad protein in stage 15 PGCs appears to be cytoplasmic. In fact, the small amount of pMad that is present earlier in stage 10–13 PGCs also appears to be predominantly cytoplasmic rather than nuclear.

The loss and subsequent reappearance of nuclear pMad in PGCs raises a number of questions. We address several of these below:

### Are Stage 10–13 PGCs Capable of Receiving and Responding to BMP Signaling?

In the period between exiting the midgut and coalescing with the SGPs to form the embryonic gonad, PGCs have little pMad protein. The low levels of pMad in stage 10–13 PGCs could be due to the absence of a potent source of BMP ligands in the surrounding somatic cells. Alternatively, stage 10–13 PGCs might not be capable of receiving BMP signals. To distinguish between these possibilities we overexpressed one of the BMP ligand family members, Dpp, using *twist-Gal4* to drive *UAS-dpp* expression in the mesoderm. As illustrated by the stage 12 *twi-Gal4/UAS-dpp* embryo in Fig. 2C and D, we found that stage 10–13 PGCs are able to transduce BMPs signals and ectopic *dpp* induces a substantial accumulation of nuclear pMad (arrows).

**Figure 4:**
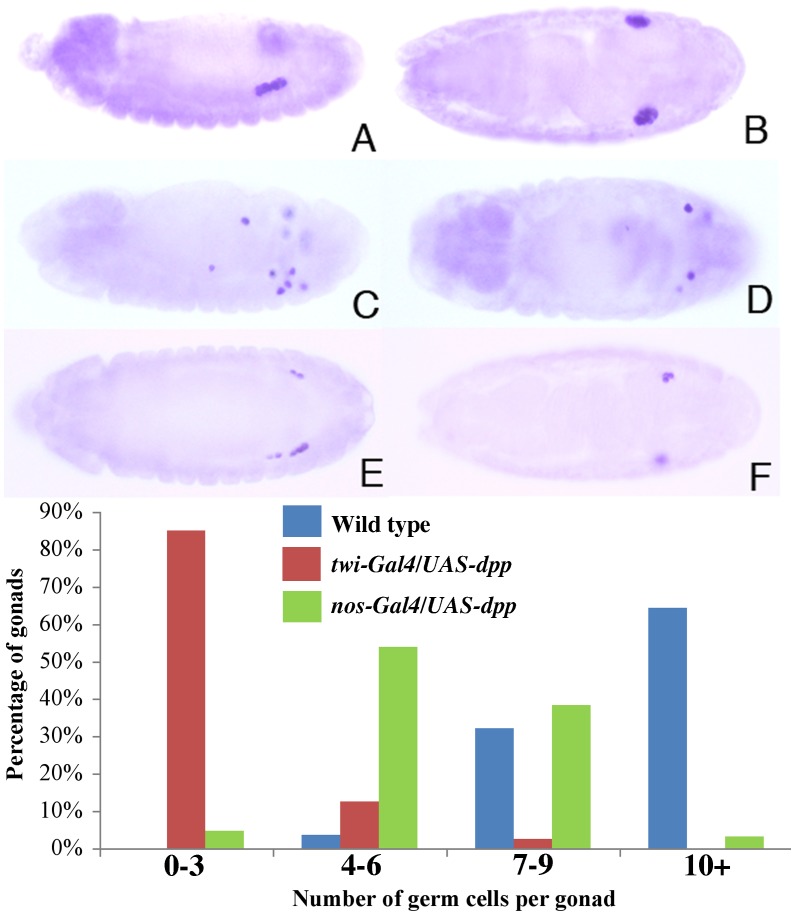
Overexpression of Dpp has deleterious effects on PGCs. **A, C, E.** Stage 13. **B, D, F.** Stage 15. **A, B.** Wild type embryos. **C, D.** *twi-Gal4/+; UAS-dpp/+* embryos. Germ cell migration defects are evident in the stage 13 embryo (C) while the gonad of the stage 15 embryo (D) has a reduced number of PGCs. Patterning defects are evident in both. **E, F.** *nos-Gal4/UAS-dpp* embryos. Both stage 13 (E) and 15 (F) embryos have fewer than normal PGCs. Only a minor germ cell migration defect is evident in (E). There are no obvious patterning defects. **G.** The number of germ cells per gonad is reduced in embryos overexpressing Dpp in the mesoderm (twi-Gal4/UAS-dpp) or in the gonad (nos-Gal4/UAS-dpp) as compared to wild type. The deleterious effects of *dpp* over-expression did not appear to be sex specific as both male and female embryo showed germ cell loss (data not shown). Embryos have a single copy of both the *Gal4* driver as indicated and the *UAS-dpp* transgene.

Since PGCs enter a “quiescent” state as soon as they are formed at the blastoderm stage, it is generally thought that they are refractory to the effects of cell- cell signaling molecules produced in the soma until the formation of the embryonic gonad. For this reason, it seemed possible that even though high levels of nuclear pMad can be generated in stage 10–13 PGCs by ectopic *dpp,* the global inhibition of Polymerase II transcription would effectively neutralize any regulatory effects of activating the BMP signal transduction cascade. However, this does not seem to be the case as we observed three unusual PGC phenotypes. First, there is a substantial reduction in the number of PGCs in the coalesced gonad (compare B and D in Fig. 4). Whereas wild type embryos have on average 12 PGCs per gonad (n = 28), there are only 3 PGCs in the coalesced gonad of stage 15 *twi-Gal4/UAS-dpp* embryos (n = 20; p<10–10). Second, there are migration defects and a significant fraction of the PGCs scatter through the embryo instead of making contact with the SGPs. This difference is shown for stage 13 embryos in Figure 4A and C. Third, many of the PGCs in the stage 10–15 *twi-Gal4/UAS-dpp* embryos have a reduced level of Vasa protein (not shown: see below). We also tested two other *Drosophila* BMP family members, *screw* and *glass bottom boat* using the *twist* driver. Unlike *dpp,* ectopic expression of *screw* had no major effect on PGCs. In the case of *gbb,* overexpression using the *twist* driver resulted in a relatively minor but statistically significant reduction in total number of germ cells (8.5 per gonad, n = 25). Though the effects are small, this observation could be significant as *gbb* is expressed in the mesoderm during mid-embryogenesis around the time when PGCs migrate through the midgut epithelial wall.

It seems likely that several factors contribute to the phenotypic effects of ectopic *dpp.* One is the induction of apoptosis. In wild type, expression of the *eiger* (egr) gene, a tumor necrosis factor ligand (TNF) homologue, is activated in stage 9 PGCs by a *dpp-dependent* mechanism [17]. *egr* then helps induce the apoptosis gene *head involution defective* (hid) in PGCs. We thus wondered if loss of PGCs seen upon exposure to excess Dpp is due to apoptosis. To assess this directly, we employed antibodies recognizing the cleaved Caspase 3 polypeptide. Accumulation of this activated form of Caspase 3 is a reliable marker of apoptosis. As can be seen in Figure S1, levels of cleaved Caspase 3 are elevated in the *twi-Gal4/UAS-dpp* PGCs as compared to wild type PGCs (compare panels D and E). Furthermore, more than 60% (16 out of 25) of total number of *twi-Gal4/UAS-dpp* PGCs were positive for cleaved Caspase 3 as opposed to 8% (2 out of 25) wild type PGCs.

### PGCs are Competent to Respond to Autocrine BMP Signals

While induction of pMad accumulation by ectopic *dpp* demonstrates that stage 10–13 PGCs are capable of receiving and responding to BMP signals, interpretation of the phenotypic effects on PGC development is complicated by the severe somatic patterning defects induced by broad *dpp* expression in the mesoderm. To reduce in so far as possible the side effects of abnormal somatic patterning on PGC development we ectopically expressed *dpp* using a *nanos- Gal4* driver (*nos-Gal4::VP16*). This driver is thought to be specific for the germline. In addition, its activation is delayed until stage 9–10 [7] whereas the *twist* driver comes on earlier in development. As might be expected from the restricted expression of the *nos* driver, we see no obvious abnormalities in somatic development in *nos-Gal4/UAS-dpp* embryos. Moreover, as illustrated in Figure 4E, there are little if any defects in germ cell migration and most PGCs are properly aligned with the SGPs in stage 13 embryos. This finding indicates that the migration defects evident with the *twist* driver are due to abnormalities in somatic patterning induced by the mesodermal expression of Dpp.

**Figure 5:**
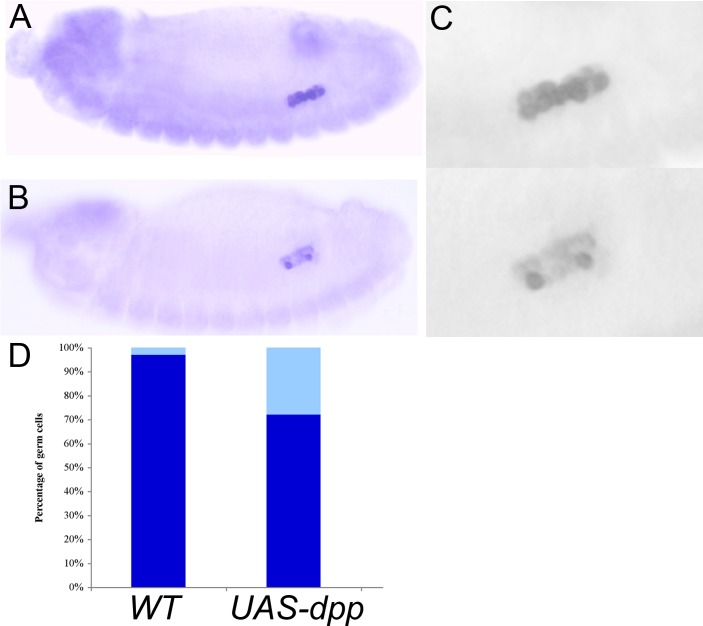
Effects of excess of BMP signaling on Vasa protein. **A.** Stage 15 wild type embryo. Gonad is tightly coalesced and all germ cells stain strongly for Vasa. **B.** Stage 15 *nos-Gal4/UAS-dpp* embryo. Most germ cells stain in this embryo only faintly for Vasa. **C.** Blow-up grey scale image of Vasa accumulation in the PGCs shown in the embryos in panels A and B. Upper panel: WT PGCs. Lower panel: *nos-Gal4/UAS-dpp* PGCs. **D.** Vasa expression in WT, *nos-Gal4/UAS-dpp* and *nos-Gal4/UAS-lwrDN* stage 15 embryos. Embryos have a single copy of both the *Gal4* driver and the *UAS* transgene as indicated. Dark blue: Percent of PGCs having normal levels of Vasa. Light blue: Percent of PGCs having an obvious reduction in the levels of Vasa compared to their sibs in the same embryo. For this analysis, Vasa levels were scored by comparing staining in the PGCs of the same embryo, not between different embryos. In a wild type embryo (processed in parallel) all PGCs have a similar and uniformly high level of Vasa protein. In contrast, when BMP signaling is upregulated, the level of Vasa protein in different PGCs in the same embryo can differ.

On the other hand, the other phenotypic effects of Dpp on PGC development are still observed with the *nos* driver. Like the *twist* driver, there is a substantial reduction in the number of germ cells in the coalesced gonad (Figure 4F). The graph in Figure 4 shows that there are only 5 PGCs per gonad in *nos-Gal4/UAS-dpp* embryos (n = 41) as opposed to 12 PGCs per gonad in wild type embryos (n = 28; p<10^−10^). Similarly, nearly a quarter of the germ cells in *nos-Gal4/UAS-dpp* embryos have noticeably reduced levels of Vasa (23% of stage 15 germ cells, n = 645; as compared to 3% wt, n = 387). This reduction can be seen in the *nos-Gal4/UAS-dpp* embryo shown in Figure 5B and in enlargements of the gonads shown in Figure 5C. Furthermore, as in the case of *twi-Gal4/UAS-dpp* combination, the germ cell loss appears to be due to apoptosis as cleaved Caspase 3 is also elevated in *nos-Gal4/UAS-dpp* PGCs (Figure S1, compare panels D and F).

Although expression of Dpp in PGCs using the *nos* driver has no obvious effects on somatic patterning, it is still possible that some critical transformation in a key somatic tissue has occurred that is too subtle to detect. To provide further evidence that it is the transduction of the *dpp* signal by the PGCs that is important we examined the effects of a constitutively active Tkv receptor, TkvA. The effects of expressing *tkvA* in the PGCs were comparable with those seen for *dpp.* On average there were 7 germ cells per coalesced gonad in *nos-Gal4/UAS-tkvA* (n = 50, p<10^10^). Moreover, as was the case for *nos-Gal4/UAS-dpp* PGCs, we also observed a reduction in Vasa accumulation in *Nos-Gal4/UAS-tkvA* (27%, n = 287).

Taken together these findings establish that stage 10–13 PGCs are competent to receive and respond to BMP signals. Moreover, it is clear that ‘too much’ BMP signaling during this stage of development has deleterious effects on PGCs, and results in a reduction in the number of germ cells that are incorporated into the embryonic gonad. Most likely this loss is due to the induction of cell death pathways by excess BMP signaling [17].

### Is BMP Signaling Important for PGC Development during Mid-embryogenesis?

While the findings described above demonstrate that PGCs have the molecular machinery needed to sense and respond to BMP signals in mid- embryogenesis, the observed effects rely on ectopic expression and thus it is entirely possible that this signaling pathway is of no significance to the development of the PGCs at this stage. To address this issue we examined the effects of mutations in the gene encoding the BMP signaling molecule *dpp*.

Properly specified PGCs have a germ cell specific organelle called the spectrosome [18], [19]. The spectrosome is first assembled in PGCs at stage 11, as the germ cells begin to migrate through the mesoderm. It is known that proper spectrosome assembly and elaboration depends upon at least one maternal component of the pole plasm, Nanos [20]. The spectrosome in the PGCs of coalesced stage 15 gonads is large and spherical in shape and contains several characteristic proteins including Alpha-spectrin and Hu li tai shao (Hts). While the size and structure of the spectrosome is similar in all of the germ cells in the newly coalesced embryonic gonad, this is not true later on in the adult gonad. Instead, a spectrosome with a size and morphology resembling that in stage 15 PGCs is only found in the subpopulation of germ cells that correspond to the germline stem cells (GSCs). During each asymmetric division of the GSCs, the structure of the spectrosome in the daughter that retains GSC identity is maintained, while in the daughter that gives rise to the gametes, the spectrosome undergoes a structural transformation. It forms a more diffuse and branched structure and bifurcates with each subsequent mitotic division of this daughter cell. Thus, the presence and morphology of the spectrosome can be used to follow the acquisition and maintenance of a “stem cell-like fate” in *Drosophila* PGCs.

**Figure 6:**
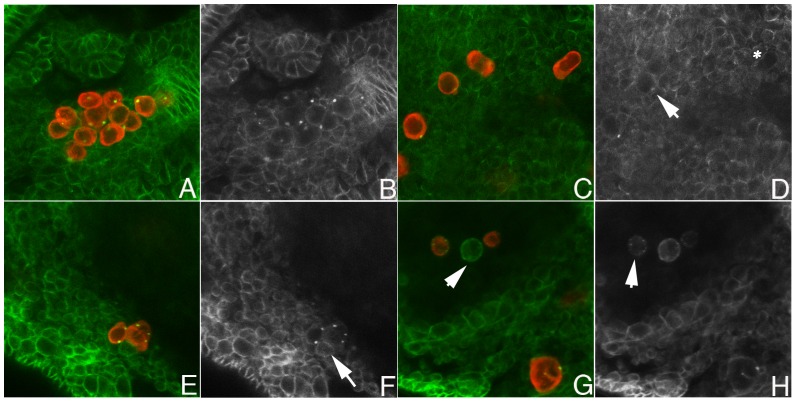
*dpp* is needed to maintain spectrosome integrity and PGC identity Panels A, C, E, and G: Vasa image in red, Spectrin imaged in green. **Panels B, D, F, and H:** Grey scale images of Spectrin. **A, B.** PGCs in stage 15 *dpp−/CyO,P<dpp>* gonads have wild type spectrosomes and normal levels of Vasa protein. The spectrosomes of stage 15–16 gonads are large well-defined spherical structures that contain Spectrin (green/grey) and, Hts (not shown). The PGCs have a spherical shape and contain high levels of cytoplasmic Vasa (red). **C–H.** *dpp−/−* embryos. **C, D.** One germ cell shows normal spectrosome morphology (arrow), while others have a diffusely labeled spectrosome (arrowhead) or lack any visible spectrosome (*). Several germ cells also show abnormal morphology. **E, F.** A cluster of germ cells. Some have normal looking spectrosome foci, while others appear to have extra spectrosome foci (arrow in F). **G, H.** One germ cell shows a complete loss of Vasa but has retained some Spectrin (arrowhead in G) while others show fragmented spectrosomes (arrow in H).

We used Spectrin and Hts antibodies to monitor spectrosomes in the PGCs of wild type and *dpp* mutant embryos. Since *dpp* is haploinsufficient, homozygous mutant *dpp* embryos were generated using a *dpp* null allele, *dpp^H46^,* and a balancer that carries two copies of *dpp.* In the experiment shown in Figure 6, we restricted our analysis to *dpp^H46^/dpp^H46^* embryos. The newly formed spectrosomes in PGCs of stage 11–12 *dpp^H46^* embryos are similar in size and morphology to wild type of the same age (not shown). However, in older embryos differences were readily apparent and the spectrosomes in *dpp^H46^* PGCs (n = 75) fell into three classes. PGCs in the first class (39%) resembled wild type and had a single large spherical spectrosomes, which stained strongly for both Spectrin (Figure 6A) and Hts (not shown). PGCs in the second class (28%) had either small diffusely stained spectrosomes (arrow in Figure 6D) or several small fragmented spectrosomes (Figure 6F & arrowhead in H). Finally, PGCs in the third class (33%) had no spectrosome and there was little if any evidence of Spectrin (* in Figure 6D) or Hts (not shown) protein speckles. In addition to these spectrosome defects other abnormalities were evident. For example, while wild type PGCs have a very regular spherical shape, the PGCs in the *dpp* mutant embryos are often deformed and irregular in appearance. Significantly, many of the *dpp^H46^* PGCs also have reduced levels of Vasa protein. In some of the PGCs, little or no Vasa could be detected (arrowhead in Figure 6G).

Since spectrosome morphology and Vasa expression are relatively normal in stage 11–12 *dpp^H46^* PGCs, it would appear that BMP signaling is not needed for spectrosome formation or the establishment of a PGC-like identity. However, the phenotypes seen as the *dpp^H46^* embryos age would argue that signaling from the soma is subsequently needed to ensure spectrosome integrity and continued growth. In addition, somatic signaling must also be important for maintaining a PGC-like identity. One caveat with these results is the fact that *dpp* is essential for dorsal- ventral patterning of the Drosophila embryo. *dpp^H46^* embryos have severe patterning defects and lack many tissues and cell types found in wild type embryos. Additionally, germ cell migration is abnormal and the PGCs never coalesce into gonads. This is likely due to a failure to properly specify the cells that form the somatic components of the gonad, the SGPs. Thus, while these findings indicate that somatic signals are critical for PGC development after stages 12–13, the gross developmental defects in the *dpp^H46^* embryos leave open the possibility that the BMP pathway is required only indirectly to generate some other somatic signaling molecule that is needed for the proper elaboration of PGC identity.

### Requirement for BMP Signaling Depends upon the BMP Receptor Tkv

**Figure 7:**
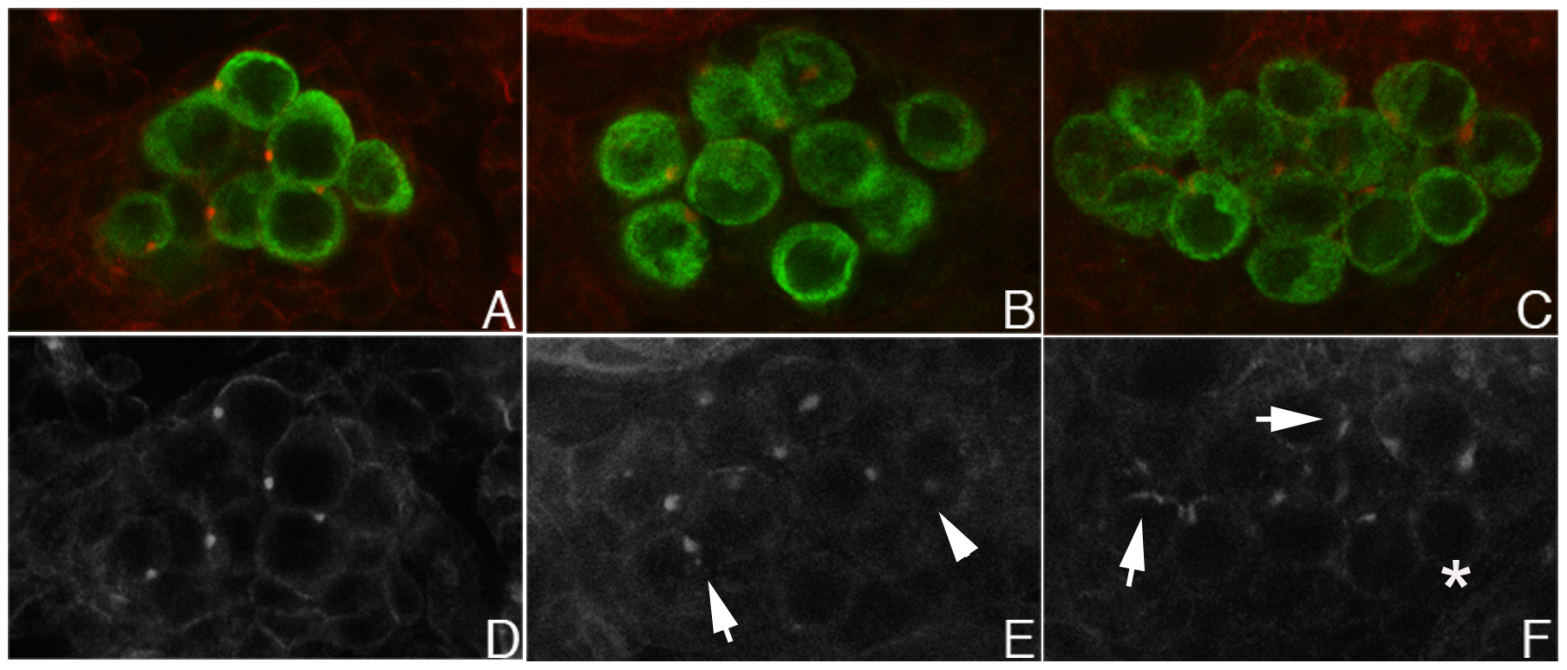
Germ cells from *tkv* germline clone mothers have spectrosome defects. Panels A–C: Stage 15 *tkvm-z+* gonads stained for Vasa (green) and Spectrin (red). **Panels D–F:** Same embryos as in A–C, except that a gray scale is used to image Spectrin. **A, D.** Germ cells exhibiting normal spectrosome morphology. Spectrosomes are spherical, well- defined and, show a strong Spectrin signal. **B, E.** Germ cells showing weak, diffuse Spectrin staining or irregularly shaped spectrosomes (arrows). **C, F.** Germ cells with fragmented (arrows) or missing (arrowhead) spectrosome.

To determine whether the requirement for BMP signaling is direct or indirect we first generated *thickveins^8^* (*tkv^8^*) female germline clones and mated the females to wild type males. The resulting progeny are compromised maternally for the *tkv* BMP receptor, but are rescued zygotically. Unlike *dpp^H46^* embryos, somatic patterning in *tkv^m^* ^−*z+*^ embryos appears to be entirely normal and the PGCs migrate to and coalesce properly with the SGPs to form an embryonic gonad. Although both maternal and zygotic *tkv* must be removed in order to completely eliminate pMad accumulation in blastoderm stage PGCs, elimination of maternal *tkv* reduces the level of nuclear pMad [9]. Since transcription is generally downregulated in PGCs until stage 9–10, it seemed possible that *tkv^m^* ^−*z+*^ PGCs would have reduced *tkv* activity compared to PGCs from wild type mothers. As would be expected the effects of reducing but not eliminating *tkv* activity are considerably less dramatic than those seen in *dpp* mutant embryos. We found that the spectrosomes in about half of the *tkv^m^* ^−*z+*^ PGCs resembled wild type (n = 50). They had the characteristic spherical shape and stained strongly for Spectrin and Hts (Figure 7A and D). On the other hand, the remaining PGCs had spectrosome phenotypes that were similar though not as severe as in *dpp^H46^* embryos. Some *tkv^m^* ^−*z+*^ PGCs have a single spectrosome, but unlike wild type are either bifurcated or have an irregularly shaped morphology resembling the spectrosomes/fusomes in differentiating germline cells in the adult gonad (arrows E and F). In other PGCs, the spectrosome is weakly labeled (arrowhead in E) fragmented (arrows in F) or missing altogether (* in F).

While the disruptions in spectrosome integrity in PGCs from *tkv* germline clone embryos were similar to those seen in *dpp* mutants, we did not observe any obvious reductions in Vasa protein accumulation in the *tkv^m^* ^−*z+*^ PGCs. This could mean that BMP signaling is required for the maintenance and elaboration of the spectrosome but that it has only an indirect role, presumably via some other BMP dependent somatic signal, in ensuring Vasa protein accumulation. Alternatively since zygotic transcription is known to provide a source of *tkv* in *tkv^m^* ^−*z+*^ PGCs [9], it is possible that Vasa accumulation is less sensitive to reduced levels of BMP signaling.

**Figure 8:**
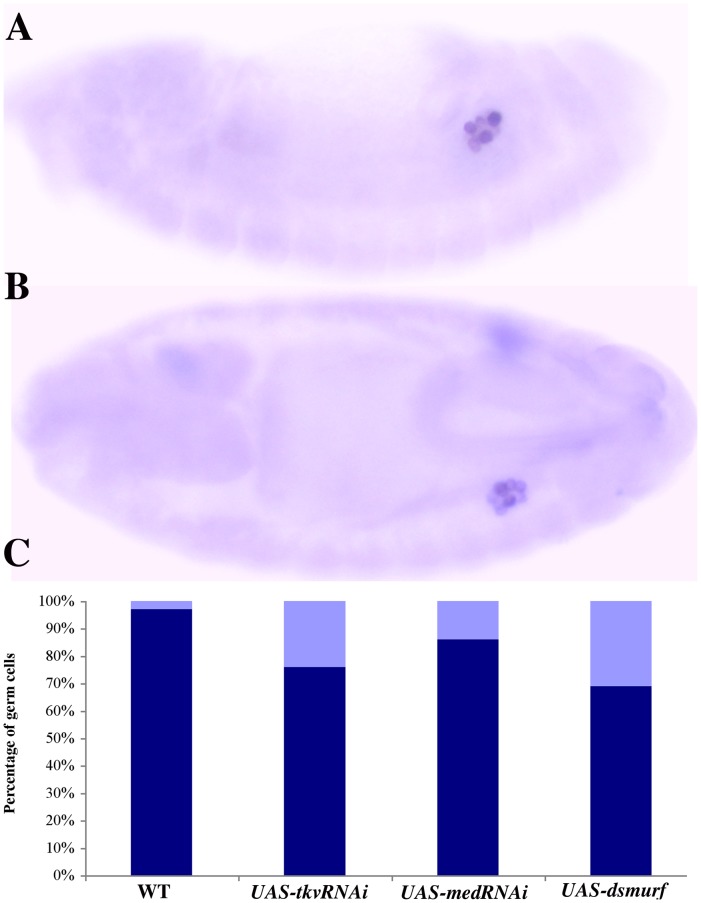
Vasa protein is lost when the ability of PGCs to respond to BMP signals is compromised. **A.** Stage 13 *nos-Gal4;UAS-dsmurf* embryo. Germ cells show unequal Vasa accumulation. **B.** Stage 15 *nos-Gal4;UAS-dsmurf* embryo. Again, germ cells show uneven Vasa accumulation. **C.** A substantial percentage of germ cells receiving insufficient BMP signaling display reduced Vasa accumulation. Dark blue: Percentage of germ cells having high levels of Vasa. Light blue: Percentage of germ cells having reduced levels of Vasa. For this analysis, Vasa levels were scored by comparing staining in the PGCs of the same embryo, not between different embryos. In a wild type embryo (processed in parallel) the PGCs have a similar and uniformly high level of Vasa protein. In contrast, when BMP signaling is compromised, the level of Vasa protein in different PGCs in the same embryo differs. In addition to the variability in Vasa protein levels shown here, a comparison with wild type embryos processed in parallel using the confocal microscope indicates that Vasa protein levels are generally reduced in PGCs compared to wild type when BMP signaling is compromised. Again, as was the case for germ cell loss when Dpp is over expressed, the reduction in Vasa levels does not appear to be sex-specific.

To further test the role of BMP signaling in these two aspects of PGC development we used *nos-Gal4* to drive the expression of a *UAS-tkvRNAi* transgene. Since the *nos* driver does not come on until stage 9–10, the RNAi knockdown would be expected to first begin compromising *tkv* activity around or after the time when the spectrosomes are beginning to assemble. In spite of this delay, the RNAi knockdown gave abnormal spectrosome phenotypes quite similar to those seen in the *tkv* germline clones. We found that 59% of the *nos-Gal4/UAS-tkvRNAi* PGCs had spectrosomes that resembled wild type, 21% had faint and fragmented spectrosomes and 21% lacked spectrosomes altogether (n = 29). Importantly, the PGCs in *nos-Gal4/UAS-tkvRNAi* embryos differ from those in *tkv^m^* ^−*z+*^ embryos in that there were obvious reductions in Vasa protein accumulation. Figure 8 shows that nearly 25% of the stage 15 *nos-Gal4/UAS-tkvRNAi* PGCs have reduced levels of Vasa. PGCs with reduced levels of Vasa protein were also observed when BMP signal transduction was compromised in the germline using *nos-Gal4* to drive the expression of RNAi specific for the pMad co-factor Medea (Figure 8). These findings, taken together with the effects of the *dpp* mutations, would argue that BMP signaling is directly required to maintain normal levels of Vasa in PGCs.

### Smurf Overexpression Disrupts PGC Development

The loss of Vasa expression coupled with the disruptions in the germline-specific organelle, the spectrosome, argue that BMP signaling is essential for maintaining a PGC-like identity during mid-embryogenesis in the period leading up the coalescence of the embryonic gonad. To provide further evidence in support of this conclusion, we used a *nos-Gal4* driver to ectopically express the ubiquitin E3 ligase, *dSmurf* [21]. The dSmurf E3 ligase has been shown to specifically interact with and ubiquitinate the activated, phosphorylated Mad protein [22]. The ubiquinated pMad is then degraded and this is thought to provide a mechanism for tempering the transcriptional response to BMP signals. When dSmurf is ectopically expressed in germ cells using the *nos-Gal4* driver, it would be expected to begin downregulating the response of the PGCs to external BMP signals only after they exit the midgut and start to reactivate transcription.

The effects of ectopic dSmurf on PGCs are even more pronounced than those seen when we partially compromised *tkv* activity. We find that only 37% of the germ cells have spectrosomes which resemble wild type, while the remaining germ cells (63%; n = 74) have fragmented, faint (16%) or missing spectrosomes (47%). Similarly, over 30% of the *nos-Gal4/UAS-dSmurf* PGCs had noticeably reduced levels of Vasa protein (Figure 8).

### Loss of Vasa under Conditions of Excess BMP Signaling

The fact that we saw loss of Vasa not only when there was too little BMP signaling but also when there was too much (see above and Figure 5) prompted us to reinvestigate the fate of PGCs under conditions of excess signaling. Significantly, we found that ectopic *dpp* had no effect on spectrosome integrity in the surviving PGCs (not shown). Thus, we suspect that the reduction in Vasa levels seen in some PGCs exposed to excess *dpp* is not due to the loss of a PGC-like identity as in seems to be the case when BMP signaling is compromised. A more likely possibility is that it is related to the death of the PGCs [17].

As a further control we also tested whether disrupting the coalescence of PGCs and SGPs into the embryonic gonad has any effect on Vasa protein accumulation. For this purpose we ectopically expressed Hmgcr (3-Hydroxy-3-Methylglutaryl Coenzyme A reductase) in the nervous system using an *elav*-*Gal4* driver [3]. This results in the production of a PGC attractant by cells in the nervous system, which leads to aberrant migration by the PGCs. As shown in shown in Figure S2, the mismigrated PGCs have normal levels of Vasa protein.

### How is BMP Signaling to the PGCs Fine-tuned?

The experiments in the previous sections indicate that PGCs require BMP signaling to maintain spectrosome integrity and a PGC-like identity in the period after they exit the mid-gut. However, we also found that excess BMP signaling during roughly this same interval has detrimental effects on PGC development including reduced Vasa, upregulation of the apoptotic protein Egr and PGC loss. The negative effects of both too little and too much BMP signaling pose a question: how is the proper balance achieved?

A possible hint as to one of the balancing mechanisms comes from a comparison of pMad localization in the PGCs of early, blastoderm stage embryos and in the coalesced gonad. While blastoderm stage PGCs have high levels of nuclear pMad, this is not true of the PGCs in the coalesced embryonic gonad. Though pMad can be detected in the nucleus at this stage, much of it seems to be localized in the cytoplasm. Miles *et al.* [23] have recently reported that BMP signaling can be downregulated in receiving cells through sumoylation of the transcription co- factor Med by a mechanism involving the *Drosophila* Ubc9 homolog Lesswright (Lwr). When Med is sumoylated in somatic cells it exits the nucleus, and if it is in a complex with pMad, it can also drag it along into the cytoplasm. This sequestration of sMed and pMad in the cytoplasm (plus the turnover of the cytoplasmic proteins) attenuates the transcriptional response of the somatic cells to BMP signals. Suggesting that such a mechanism could operate in the embryonic germline, Hashiyama *et al.* [24] found that mRNAs encoding *lwr* (as well as other sumoylation pathway genes) are greatly enriched in PGCs. Although their subsequent studies uncovered a sex-specific function for *lwr* in PGC development [25], the fact that PGCs in the coalesced gonad (of both sexes) have high levels of cytoplasmic pMad suggests that Lwr might also be responsible for reining in the response to BMP signals.

If one of the functions of *lwr* in the germline is to attenuate BMP signaling, then reducing *lwr* activity should alter the pattern of accumulation of the downstream effectors. To test this prediction we used *nos-Gal4* to drive the expression of a transgene, *UAS-lwrDN,* that encodes a dominant negative Lwr protein. The dominant negative Lwr protein should interfere with *lwr* activity and mimic the upregulation of the signaling pathway that is seen when *dpp* is ectopically expressed using a germline driver. On the other hand, since the inhibition of *lwr* activity is expected to require the accumulation of sufficient quantities of LwrDN, the effects on pMad accumulation are likely to smaller compared to those observed when Dpp is expressed using the same *nos-Gal4* driver. A comparison of pMad accumulation in *nos-Gal4/UAS-lwrDN* PGCs with that in *nos-Gal4/UAS-dpp* and wild type PGCs is shown in Figure 3. We find that the accumulation of pMad in the nucleus of *nos- Gal4/UAS-lwrDN* (Figure 3C) PGCs is intermediate between the relatively low level seen in wild type (Figure 3A) and the high level observed in *nos-Gal4/UAS-dpp* PGCs (Figure 3B). LwrDN also affects Med accumulation; however, in our experiments it seems to increase the overall levels of both cytoplasmic and nuclear Med in the PGCs rather than just promote nuclear accumulation (not shown).

**Figure 9:**
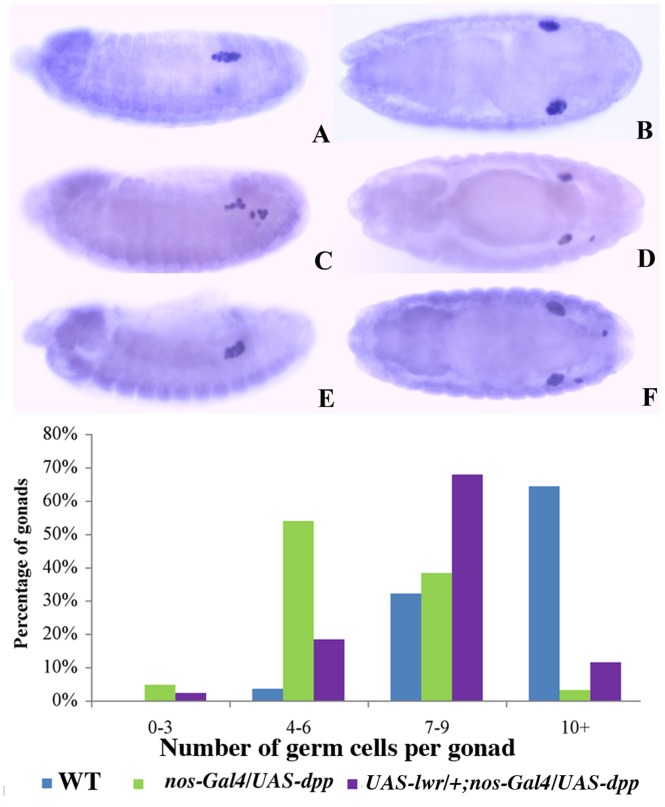
*lesswright* downregulates BMP signaling in PGCs. Panels **A, B, C:** Stage 15 embryos. **A:** Wild type. **B:** *nos-Gal4/UAS-dpp* embryo showing reduction in PGCs. **C:** *UAS-lwr/+;nos-Gal4/UAS-dpp* embryo showing intermediate number of PGCs indicating partial rescue of PGC loss phenotype observed in *nos-Gal4/UAS-dpp* embryos. *UAS-lwr/+; nos-Gal4/UAS-dpp* stage 15 embryos have an average of 8 PGCs per gonad as compared to 5 PGCs/gonad for *nos-Gal4/UAS-dpp* and 12 PGCs/gonad for wild type. All transgenic embryos had a single copy of the *Gal4* driver.

These findings would argue that in addition to its sex-specific germline functions [25], *lwr* also acts to dampen BMP signaling in PGCs. In this case, it should be possible to suppress, at least partially, the phenotypic effects of too much BMP signaling on PGC development by upregulating *lwr* activity in germ cells. To test this prediction we simultaneously expressed *dpp* and wild type *lwr* in PGCs using the *nos-Gal4* germline driver. Figure 4F and Figure 9B show that when *dpp* is expressed in the germline, the number of PGCs in the coalesced gonad is reduced to around 5 compared to 12 for wild type embryos. Although co-expression of *lwr* with *dpp* does not restore the number of PGCs to wild type levels, there is a clear shift in the distribution of PGCs/gonad when the activity of *lwr* is increased (Figure 9A–C and the accompanying graph). In this case the average number of PGCs in the gonad is around 8 (n = 43, p<108).

## Discussion

The deleterious effects of excess BMP signaling in the period leading up to gonad formation indicate that embryonic PGCs are not only able to sense but can also respond to somatic signals even before they become tightly associated with the somatic components of the gonad. Since PGCs rely on somatic signals to guide their migration towards the SGPs, this finding is not all that surprising; however, our experiments also indicate BMP signaling is required for proper PGC development. A role for soma-germline signaling in PGC development is unexpected since this process, at least until embryonic gonad coalescence, was thought to depend entirely on cell autonomous factors deposited in the posterior pole plasm during oogenesis [3], [4], [6].

One feature that sets germ cells apart from surrounding somatic cells is a specialized cytoplasmic organelle called the spectrosome. Wawersik and Van Doren [20] have shown that a cell autonomous process, which depends upon maternally deposited *nos,* initiates the assembly of spectrosomes soon after the PGCs exit the midgut. These cytoplasmic structures then grow in size until after the coalescence of the embryonic gonad. Consistent with their findings, BMP signaling does not appear to be required for the initial formation of the spectrosomes. However, we found that the subsequent maintenance of spectrosome integrity in stage 13–16 PGCs depends upon the BMP signaling pathway. When BMP signaling is compromised, the spectrosomes cease growing, and instead begin to fragment and disappear completely. The spectrosomes are not the only marker of germ cell identity that depends upon BMP signaling. We also found that PGCs are unable to maintain normal levels of the germline specific marker Vasa when BMP signaling is compromised. The most dramatic effects are evident in *dpp* mutant embryos, where we observe PGCs that have little or no Vasa protein. However, loss of the Vasa germline marker is also evident when the ability of the PGCs to respond to external BMP signals is perturbed by partially compromising the activity of the *tkv* receptor or the downstream transcription factors Mad and Med, or by overexpressing the negative regulator dSmurf. While the involvement of Mad and Med and their regulators would argue that BMP signals impact PGC development by modulating transcription, it is also possible that the signaling pathway acts via other mechanisms.

One plausible interpretation of the coupling of the spectrosome and Vasa phenotypes is that PGCs need BMP signals to maintain their PGC-like identity. Assuming that this interpretation is correct, an important question is whether BMP signals have an instructive role with respect to PGC identity, or instead function only to reinforce or sustain the instructive activities of the cell autonomous factors in the pole plasm that are incorporated into the germ cells when they are formed. Transplantation experiments by Illmensee and Mahowald [26] (see also [27]) have suggested that localized maternal factors are sufficient to specify fully functional PGCs. If this is the case, then the function of BMP signaling must be restricted to reinforcing the instructive activities of these cell autonomous factors. On the other hand, it is possible that the transplanted PGCs are also subjected to external BMP signals at critical junctures during their specification. Moreover, there is evidence that PGCs need to respond to instructive signals from the soma in order to establish their proper identity. One example would be sex-determination in the germline, which is dictated in large part by somatic signals [28]–[30]. Interestingly, recent studies by Casper and Van Doren [31] indicate that these signals begin activating the sex- specific expression of genes in PGCs as the embryonic gonad coalesces. This is just slightly later than when we begin seeing effects on PGCs’ fate when BMP signaling is compromised. While further studies will be required to determine if BMP signaling has an instructive role, there is precedent for BMP signals orchestrating the specification of the germline. For example, in mammals maternally derived cell autonomous factors play no role in PGC specification. Instead, external BMP signals from the extraembryonic ectoderm have the instructive function and can induce *de novo* the formation of PGCs from the proximal epiblasts [32]–[34]. Interestingly, among the genes induced in mouse PGCs by BMP signaling is Nanos3. Like BMP signaling in flies, one of the functions of Nanos3 seems to be in maintaining nascent PGCs [35]. This is not the only similarity between flies and mammals. Childs et al [36] recently reported that the localization of pSMADs in human fetal ovary germ cells changes from being predominantly nuclear to mostly cytoplasmic as development proceeds. They also found that excess BMP signaling induces PGC apoptosis.

Like the PGCs in stage 13–15 embryos, the GSCs in the adult gonad also require external BMP signals. However, the effects of BMP signaling in each case seem to be somewhat different. In the adult, BMP signals function as a proximity switch to control GSC identity [12], [37] When the GSCs are close to the source or the signaling levels are otherwise elevated, the germ cells retain GSC identity even when they divide. When they are farther from the source, or signaling is otherwise compromised, the germ cells begin differentiating into gametes. Concomitantly, spectrosome morphology changes and the levels of Vasa and other proteins that are enriched in the stem cells (e.g., Sex-lethal and Nanos) are reduced. Critically, in both gain and loss of BMP signaling the adult GSCs retain their germline identity and follow completely normal developmental paths for these cells. In contrast, too much or too little BMP signaling appears to have a more far reaching effect on the ultimate fate of PGCs. PGCs are lost probably through Eiger upregulation and apoptosis when signaling levels are too high [17]. Conversely, when signaling levels are too low the PGCs are apparently not only unable to maintain their identity but also don’t follow a normal “germline” differentiation program. Interestingly, Sato et al [38] have shown that germ cells in the larval gonad respond to BMP signals in a manner that is also somewhat different from what is observe in either the adult or in the embryo. They found that excess *dpp* appears to induce larval PGCs to proliferate without any evidence cell death, while insufficient *dpp* seemed to reduce proliferation, but did not appear to promote cystocyte differentiation. This would suggest that PGCs must gradually acquire all of the properties of GSCs, at least with respect to BMP signaling, in the period between the coalescence of the embryonic gonad and the late larval stage. It clearly will be of interest to understand how this transition takes place and what role other signaling pathways, soma-germline interactions in and outside of the stem cell niche, or even the determination of sexual identity might play in fully converting PGCs to GSCs.

In the adult gonad, the BMP ligands essential for maintaining GSC identity are produced by somatic cells associated with the stem cell niche [10]. However, the source(s) of BMP ligands that signal to the PGCs in stage 13–16 embryos remains to be determined. While we were able to detect expression of a *dpp: lacZ* reporter in mesodermal cells close to the migrating PGCs, the identity of these cells is unknown, and it is unclear whether they are actually responsible for signaling to the PGCs. In coalesced gonads, there are several cells that express the *dpp* reporter and are in sufficiently close proximity that they could be the source of BMP signals at this stage; however, these cells do not seem to be typical SGPs and their identity is unknown.

Whatever the source of the somatic signaling cells, there are mechanisms in place in PGCs to help them adjust their response to external BMP signals. One mechanism is the sumoylation of Med. We find that interfering with the activity of the Ubc9 homolog, *lwr,* increases pMad levels in PGCs. By contrast, it is possible to suppress the deleterious effects of excess Dpp by overexpressing Lwr in PGCs. One interpretation of the effects of manipulating Lwr activity is that BMP signaling levels must be appropriately calibrated by the PGCs. In order for them to survive, there must not be too much BMP signaling. On the other hand, if there is too little signaling, PGCs will not be able to properly maintain their identity. In this context, it should be noted that Hashiyama *et al.* [25] have shown that *lwr* has a female-specific function in PGCs. When *lwr* activity is compromised by overexpressing the LwrDN protein, they found that female, but not male PGCs die. The sex-specific effect of compromising *lwr* in germ cells raises the question of whether gain or loss of BMP signaling also has sex specific consequences. Though we confirmed their findings with respect to over expression of LwrDN, we did not detect any obvious sex-specific differences in the assay we used to analyze the impact of BMP signaling on PGC development. It is, however, entirely possible that the BMP signaling pathway has both sex non-specific functions in PGC development as well as yet undiscovered sex-specific functions. Further studies will be required to assess this possibility.

## Materials and Methods

### Fly Stocks and Culture

Flies were grown at room temperature (22°C) on standard medium similar to the current “standard media” listed on the Bloomington stock center website. The following stocks were used for misexpression analysis: *twist-Gal4* (stock number: BL-914 and BL-2517), *nanos-Gal4::VP16* (stock number: BL-7303), *UAS-dpp* (stock number: BL-1486), *UAS-screw* (obtained from Kavita Arora/Rahul Warrior labs), *UAS-medRNAi* (stock number: BL-31928), *UAS-tkvRNAi* (stock number: BL-35166 and 31040), *UAS-madRNAi* (stock number: BL-31315), *UAS-dsmurf, UAS-lwrDN* (stock number: BL-9318), and *UAS-lwr* (stock number: BL-9324). Typically, virgin females homozygous for the *Gal4* transgene were mated with males carrying the *UAS* transgene [39]. For the mutant analysis, *dpp^H46^* was used. Germline clonal analysis was performed as described in Chou and Perrimon,1992) using *FRT-tkv^8^* allele stock obtained from Chip Ferguson’s lab. Females carrying germline clones were mated with males of the appropriate genotype [40]. Embryos from these crosses were fixed and stained for subsequent analysis. (The genotypes were identified using either ß-Galactosidase or anti-GFP antibodies as described below). *UAS*-*gbb* and *UAS*-*tkv*A (constitutively activated form of the receptor, (TKV Q253→D) stocks were provided by Michael O'Connor and Chip Ferguson’s labs respectively.

### Immunohistochemistry

The stainings were performed essentially as described in Deshpande et al (1995) [41]. Embryos were fixed for 20 min in 3.7% formaldehyde/PBS: heptane and devitellinized in heptane: methanol for staining with the primary antibodies: Conjugated Secondary antibodies were obtained from Molecular Probes. Embryos were mounted in AquaPolymount (Polysciences, Inc.). Images were collected using a Zeiss LSM 510 confocal microscope and Adobe Photoshop was used to generate the final figures. The microscope settings including the gain and the laser intensity were maintained constant for the duration of a complete experiment after the initial standardization to minimize the variability. In each imaging experiment both experimental and control embryos were imaged under the same conditions. It should be noted that while analyzing the spectrosomes different planes of focus had to be used to visualize and assess the integrity of the structure. (While the figure panels represent a single section in most instances, quantitation was performed to assess the extent of defects as described in the results section). Vasa antibody was either a rat or rabbit polyclonal used at 1∶500 dilution (from Paul Lasko). pMad is a rabbit polyclonal antibody and was used at 1∶500 dilution. (The antibody was a kind gift from Ed Laufer). Spectrin mouse monoclonal antibody (DSHB) was used at 1∶5 dilution, and Hu-li tai shao mouse monoclonal antibody (DSHB) was used at 1∶5 dilution. ß-Galactosidase antibody was either a rabbit polyclonal purchased from Cappel (used at 1∶1000 dilution) or a mouse monoclonal antibody from Developmental Studies Hybridoma Bank (used at 1∶10 dilution). GFP antibody is a rabbit polyclonal purchased from Torrey Pines Biolabs (used at 1∶1000 dilution).

## Supporting Information

Figure S1 **Ectopic expression of the BMP ligand, DPP, induces apoptosis.** Embryos of the specified genotypes were double stained with Vasa and cleaved Caspase 3 antibodies. Panels A and D: *wild type*, Panels B and E: *twi-Gal4/UAS-dpp*, Panels C and F: *nos-Gal4/UAS-dpp*. Panels A-C show composite images of Vasa (red) and cleaved Caspase 3 (green) whereas panels D-F show just a gray scale image of the cleaved Caspase 3.
(TIF)

Figure S2 **Vasa levels are unaffected among the mismigrated PGCs.** Wild type and *elav-Gal4/UAS-hmgcr* embryos were stained with Vasa antibody (imaged in grey scale). Panel A: Wild-type. Panel B and C: *elav-Gal4/UAS-hmgcr*. As reported previously ectopic expression of *hmgcr* in the nervous system using the *elav-Gal4* driver induces PGC migration defect. Shown in panels B and C are PGCs from the same embryo. The PGCs on one side of the embryo that are shown in panel B have coalesced into normal looking gonad. The PGCs on the other side of the embryo are scattered from the effects ectopic Hmgcr. In both cases, the levels of Vasa protein are similar in all PGCs and equivalent to that seen in wild type PGCs.
(TIFF)
