# Supplementary figures and images for: BMP Signaling and the Maintenance of Primordial Germ Cell Identity in Drosophila Embryos
Source: PLoS One. 2014 Feb 14;9(2):e88847. doi: 10.1371/journal.pone.0088847 (PMC3925178; doi:10.1371/journal.pone.0088847)

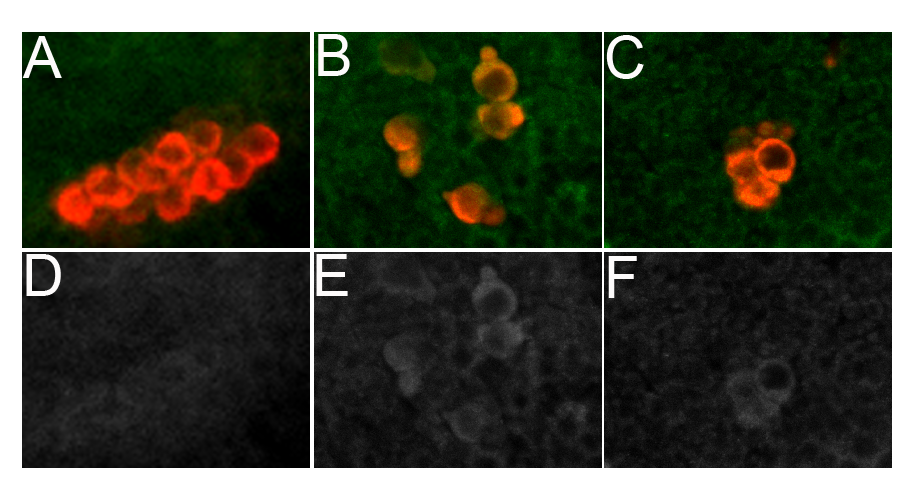

Supplement: Figure S1 — Ectopic expression of the BMP ligand, DPP, induces apoptosis. Embryos of the specified genotypes were double stained with Vasa and cleaved Caspase 3 antibodies. Panels A and D: wild type, Panels B and E: twi-Gal4/UAS-dpp, Panels C and F: nos-Gal4/UAS-dpp. Panels A-C show composite images of Vasa (red) and cleaved Caspase 3 (green) whereas panels D-F show just a gray scale image of the cleaved Caspase 3. (TIF) [file pone.0088847.s001.tif]

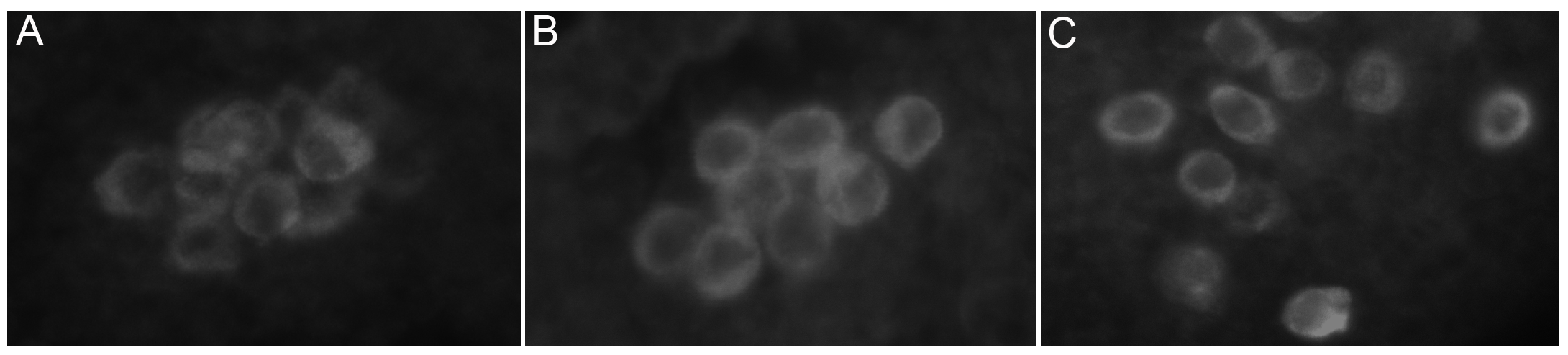

Supplement: Figure S2 — Vasa levels are unaffected among the mismigrated PGCs. Wild type and elav-Gal4/UAS-hmgcr embryos were stained with Vasa antibody (imaged in grey scale). Panel A: Wild-type. Panel B and C: elav-Gal4/UAS-hmgcr. As reported previously ectopic expression of hmgcr in the nervous system using the elav-Gal4 driver induces PGC migration defect. Shown in panels B and C are PGCs from the same embryo. The PGCs on one side of the embryo that are shown in panel B have coalesced into normal looking gonad. The PGCs on the other side of the embryo are scattered from the effects ectopic Hmgcr. In both cases, the levels of Vasa protein are similar in all PGCs and equivalent to that seen in wild type PGCs. (TIFF) [file pone.0088847.s002.tif]
